# Reconstruction of a genome-scale metabolic model for *Actinobacillus succinogenes* 130Z

**DOI:** 10.1186/s12918-018-0585-7

**Published:** 2018-05-30

**Authors:** Bruno Pereira, Joana Miguel, Paulo Vilaça, Simão Soares, Isabel Rocha, Sónia Carneiro

**Affiliations:** 1 SilicoLife Lda, Rua do Canastreiro 15, 4715-387 Braga, Portugal; 2 CEB - Centre of Biological Engineering, University of Minho, Campus de Gualtar, 4710-057 Braga, Portugal; 3 Instituto de Tecnologia Química e Biológica António Xavier, Universidade Nova de Lisboa (ITQB-NOVA), Oeiras, Portugal

**Keywords:** Genome-scale metabolic reconstruction, Constraints-based flux analysis, Succinic acid fermentation

## Abstract

**Background:**

*Actinobacillus succinogenes* is a promising bacterial catalyst for the bioproduction of succinic acid from low-cost raw materials. In this work, a genome-scale metabolic model was reconstructed and used to assess the metabolic capabilities of this microorganism under producing conditions.

**Results:**

The model, *i*BP722, was reconstructed based on the functional reannotation of the complete genome sequence of *A. succinogenes* 130Z and manual inspection of metabolic pathways, covering 1072 enzymatic reactions associated with 722 metabolic genes that involve 713 metabolites. The highly curated model was effective in capturing the growth of *A. succinogenes* on various carbon sources, as well as the SA production under various growth conditions with fair agreement between experimental and predicted data. Calculated flux distributions under different conditions show that a number of metabolic pathways are affected by the activity of some metabolic enzymes at key nodes in metabolism, including the transport mechanism of carbon sources and the ability to fix carbon dioxide.

**Conclusions:**

The established genome-scale metabolic model can be used for model-driven strain design and medium alteration to improve succinic acid yields.

**Electronic supplementary material:**

The online version of this article (10.1186/s12918-018-0585-7) contains supplementary material, which is available to authorized users.

## Background

*Actinobacillus succinogenes* is a gram-negative facultative anaerobic bacterium and is one of the major natural producers of succinic acid (SA). It can grow on a broad range of substrates, including arabinose, cellobiose, fructose, galactose, glucose, lactose, maltose, mannitol, mannose, sucrose and xylose, producing a mixture of by-products (e.g., SA, formic acid (FA), acetic acid (AA), and ethanol (EtOH)) as the main by-products [1, 2]. Its tolerance to high sugar concentrations (up to 160 g.L^− 1^ of glucose [3]) and high levels of organic acids [4], as well as its capnophilic nature [5], make this microorganism potentially interesting for the production of SA at the industrial scale. High-titer succinate production using low-cost feedstocks like cane molasses or corn straw has been obtained [2, 6–15]; however, significant amounts of other organic acids were also produced, increasing the downstream processing costs, which makes this bioprocess less competitive. Currently, SA is mainly produced from petrochemical feedstocks through the hydrogenation of maleic acid or maleic anhydride [16]. However, the bio-based production using low pH yeast fermentation [17–19] or anaerobic fermentation using bacteria [20–23] has been successfully implemented by companies like Myriant [24], BASF [25] or BioAmber [26], offering economically and ecologically attractive alternatives to the conventional petro-based SA production [27–29]. Some examples of SA producing systems are given in Table 1, including naturally-producing bacteria like *Basfia succiniciproducens* and *Mannheimia succiniproducens* and genetically engineered organisms such as *E. coli* or *S. cerevisiae*. So far, natural producers appear to outperform most engineered strains, but developments in strain design and fermentative processes are expected to promote the production of bio-based SA by metabolically engineered microorganisms. For instance, *M. succiniproducens* has been metabolically engineered by removing competing pathways, resulting in an increase in the SA yield from 0.45 to 0.76 g of SA *per* g of glucose [30].

**Table 1:** Some examples of SA bio-production systems using natural producers or metabolic engineered organisms

Organism	Genetic modifications	Culture conditions	Carbon sources (CS)	SA Yield (g.g_CS_^−1^)	References
*B. succiniciproducens*		Anaerobic, continuous	Glycerol	1.02	[21]
*M. succiniproducens*		Anaerobic, batch	Glucose	0.59	[71]
*M. succiniproducens*	– Deletion of *ldh*A*, pfl*B*, pta,* and *ack*A genes	Anaerobic, fed-batch	Glucose	0.76	[30]
*A. succinogenes*		Anaerobic, continuous	Xylose	0.80	[63]
*A. succinogenes*		Anaerobic, batch	Glucose	0.74	[72]
*A. succinogenes*		Anaerobic, repeated fed-batch	Glucose	0.88	[31]
*E. coli*	– Deletion of *pfl*B, *ldh*A, *ppc* genes – Heterologous expression of pckA from *B. subtilis*	Anaerobic, batch	Corn stalk hydrolysate	1.02	[43]
*E. coli*	– Deletion of *iclR*, *icd*, *sdhAB*, *ackA*-*pta*, *pox*B – Heterologous expression of *pepc* gene from *Sorghum vulgare*	Aerobic, batch	Glucose	0.72	[73]
*S. cerevisiae*	– Deletion of *sdh3*, *ser3*, *ser33* – Overexpression of *ICL1*	Aerobic, batch	Glucose	0.05	[37]
*S. cerevisiae*	– Deletion of *pdc1, pdc5, pdc6*, *fum1*, *gpd1* – Overexpression of pyc2, mdh3, fumC and frds1	Aerobic, batch	Glucose	0.14	[19]

Mass yields are given in g.g^− 1^ of carbon source (CS)

Under optimized conditions, the *A. succinogenes* wild-type strain is able to produce up to 98 g.L^− 1^ of SA with an approximate yield of 90% (*w*/w) on glucose [31]. The optimization of bioprocesses has proven to further increase SA production by using high concentrations of carbon dioxide (CO_2_) and/or hydrogen (H_2_) [32, 33]. Other studies have shown that the redox state of the fermentation broth affects SA production, which can be improved by manipulating the supplementation of oxidant and reducing agents [34, 35]. Yet, due to the accumulation of other fermentative by-products, SA yields are still far below the maximum theoretical yield of 1.12 g.g^− 1^ of glucose consumed (Y_SA/Glc_) [36]. A comprehensive understanding of the metabolism and the phenotypic responses to environmental perturbations is a major step for developing efficient bioprocesses for SA production.

Genome-scale metabolic models (GSMMs) have proven to be powerful tools for understanding and re-designing the metabolism of microbial strains. For instance, the optimization of SA production in *E. coli* or *S. cerevisiae* has been achieved by applying metabolic engineering strategies supported by *in silico* modelling of metabolic networks [37–40]. There are three main pathways for SA biosynthesis, including the tricarboxylic acid (TCA) cycle in the oxidative direction, the glyoxylate shunt and the reductive TCA pathway [41]. Typically, under aerobic conditions, either the oxidative TCA cycle or the glyoxylate shunt can be used for SA production and several studies have used both *S. cerevisiae* and *E. coli* to exploit these metabolic pathways [38, 40]. The redirection of the carbon flux through the glyoxylate shunt provides some advantages over the oxidative TCA cycle, mainly because the decarboxylation of isocitrate to succinyl-CoA leads to carbon loss [40] and, in the case of *S. cerevisiae*, the SA channelling from the mitochondria to cytosol is avoided as TCA cycle enzymes are located in mitochondria. However, if a reductive TCA pathway is used, a 2-fold maximum theoretical yield (2 mol.mol^− 1^ of glucose) can be achieved compared to the oxidative route (1 mol.mol^− 1^ of glucose) [41]. Many organisms, such as *E. coli* and *S. cerevisiae* have been tested for SA production under anaerobic conditions using the reductive branch of the TCA cycle [38, 42]. However, reducing power limitations (i.e. NADH) or redox balance issues have shown to have an impact on the final SA yields [41]. Metabolic engineering strategies driven by *in silico* modelling may allow to overcome these disadvantages, both under aerobic and anaerobic conditions. Strategies for increasing energy and/or cofactor pools [43–45], to overcome enzyme limitations [46] or to decrease by-product generation [37] are just a few examples that have been exploited [47].

*A. succinogenes* produces SA anaerobically through the reductive branch of the TCA cycle (i.e., C4 pathway) using fumarate as the final electron acceptor, which makes this metabolic branch highly dependent on the redox state of cultures. Phosphoenolpyruvate (PEP) node controls the amount of flux that is directed towards the C4 and C3 pathways, adjusting the level of fermentative products generated by each pathway. Other metabolic nodes like oxaloacetate (OAA) and malate (MAL) have shown to link the C4 and C3 pathways via decarboxylating enzymes, i.e. oxaloacetate decarboxylase and the NADPH-dependent malic enzyme, respectively. The split of carbon flux at these branching points is largely influenced by various factors, such as the availability of CO_2_, pH or carbon sources. It has been previously shown that an increase in the concentration of dissolved CO_2_ in the fermentation broth through the supplementation of magnesium carbonate [48] or sodium bicarbonate [32] can promote an increase in the carbon flow toward the C4 pathway, thus increasing the production of SA. The presence of carbonic anhydrases (putative coding gene, Asuc_1199), which interconverts CO_2_ and bicarbonate (HCO_3_^−^), may also contribute to increase CO_2_ fixation [12, 49].

Here, a GSMM (named *i*BP722) representing a wide range of metabolic capabilities of *A. succinogenes* 130Z is proposed. The model allows predicting and analysing the impact of stoichiometric and physiological constraints known to apply at steady-state conditions. Although the central carbon metabolism of *A. succinogenes* 130Z has been comprehensively described [32, 33, 50, 51], the overall representation of metabolic pathways associated with various catabolic and anabolic capabilities of the organism are now made available. The biosynthetic pathways for vitamins, cofactors and other biomass building blocks are described, as well as respiratory and energy consuming assimilatory pathway. This model provides a detailed insight on the metabolism of *A. succinogenes* that can be systematically explored to improve the bioproduction of SA.

## Methods

### Metabolic functional annotation

**Fig. 1:**
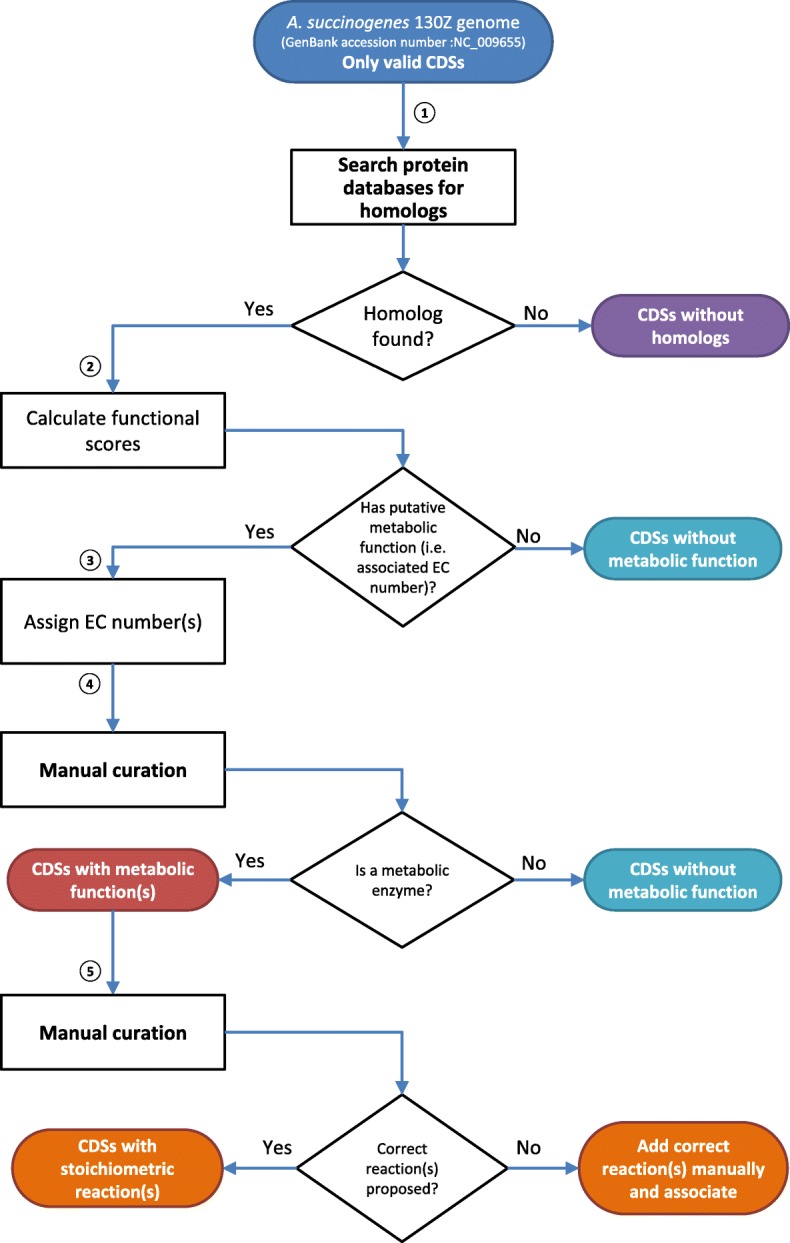
Metabolic functional annotation of CDSs. The implemented framework uses an automated annotation tool coupled with manual curation procedures that allows the assignment of metabolic functions to CDSs of a particular genome. The pipeline comprises five main steps: (1) homology search CDSs against databases like UniProt; (2) computation of functional scores based on similarity; (3) assignment of putative metabolic function(s); (4) manual curation of functions; and finally (5) association of metabolic reactions

The complete genome sequence of *A. succinogenes* 130Z (GenBank accession number NC_009655) [50] was used for the functional annotation of genes based on homology searching methods. The annotated genes with potential metabolic roles were manually inspected and associated with the corresponding coding enzyme(s) and biochemical reaction(s). An internally developed platform was used to compute, assign and curate gene metabolic functions. This platform couples automated annotation tools with manual curation procedures that allows the assignment of metabolic functions to coding sequences (CDSs) of a particular genome. The pipeline consists in five main steps (see Fig. 1):

1. The application of homology search tools like BLAST [52] and HMMER [53] against sequence databases, such as UniProt [54] to find the best alignment between sequences. A ranked list of hits with the most significant matches to each query is obtained with the respective information, including scores and protein features that may contain Enzyme Commission (EC) numbers.
2. The computation of “functional scores” based on BLAST and HMMER scores. As the range of enzymatic functions attributed to each CDS can vary from tool to tool, a second score was computed, i.e. so-called functional score, in order to propose the best candidate metabolic functions. Besides BLAST and HMMER scores, a functional score was computed similarly to the method used by Merlin [55], which is based on the frequency of the EC number in the homology hits and on the taxonomy distance between the target strain and other strains within hit results. This weighed functional scores range between 0 and 1 (1 corresponding to a high confidence score).
3. Assignment of putative metabolic function(s). EC numbers associated with the highest functional score are automatically attributed to each CDS, as well as the corresponding metabolic reaction(s) from an internal reactions database.
4. The assignment of metabolic functions to each CDS is manually revised, as automatic assignments may fail when more than one high scoring EC number is found and/or EC number(s) are incorrectly associated in databases. Therefore the user is allowed to inspect all putative assignments and select the most appropriate or modify the functional assignment, which can be based on previous knowledge or other assumptions defined by the user. Furthermore, more than one CDS can be associated to the same EC number, which in many cases consists of subunits of the same multimeric enzyme. The classification of multimeric or monomeric subunits is also defined at this stage using an internal database.
5. After validation of functional assignments and enzyme subunits, the association of metabolic reactions is carried out based on the associated EC number(s) and/or previous knowledge using as a reference an internal reactions database. This step is perhaps the most critical during the reconstruction of the metabolic network, as it will define the set of stoichiometric reactions that characterize a specific organism.

To note that this annotation pipeline is flexible enough, such that unassigned CDSs in step (3) can be later reviewed, particularly during the gap filling process.

### Construction of the metabolic network

The GSMM was initially constructed by compiling the annotated metabolic genes and their corresponding coding enzyme(s) and biochemical reaction(s). Additionally, spontaneous and transport reactions from databases like KEGG [56] and Transporter Classification Database (TCDB) [57] were added. Some metabolic reactions, although not associated with genes, were also included due to evidences found in literature. The preliminary draft model was then processed to:

- Identify metabolic gaps (or missing reactions) that either consume or produce isolated (or dead-end) metabolites within the metabolic network. Then, each dead-end metabolite was inspected to search for metabolic reactions that consume, produce or transport this metabolite. Typically, MetaCyc or KEGG databases were used for gap-filling, i.e. to identify the sets of biochemical reactions that link each dead-end to a metabolite in the network. When several alternatives are found, a manual inspection is required and sequence-based homology searches using one or more amino acid sequences collected from potential candidates are used to find the most likely reactions set in the metabolic network of *A. succinogenes* 130Z.
- Infer and correct the mass and charge balance of biochemical reactions. Stoichiometric coefficients of compounds in reactions are corrected, such that the reaction is balanced for mass and charge, usually by adding missing protons or water molecules.
- Identify and correct the reversibility of reactions based on their thermodynamic properties and information found in literature. Databases like MetaCyc [58] and tools like eQuilibrator [59] were used.
- Include a biomass reaction representing the basic macromolecular composition of *A. succinogenes* in terms of proteins, DNA, RNA, lipopolysaccharide (LPS), phospholipids, peptidoglycan, glycogen and cofactors and vitamins (CAV). The synthesis of each macromolecule was also represented by individual reactions considering the building blocks molar composition. For instance, the synthesis of one gram of protein was calculated based on the average amino acids composition using the set of encoded proteins of the *A. succinogenes* 130Z genome, according to the methodology proposed in [60]. The synthesis of other cellular components, like CAV, was calculated assuming that each small molecule is equally present in one gram of CAV. Detailed information on the biomass composition can be found in Additional file 1.

### Constraints-based flux analysis

Basic stoichiometric modelling methods, such as parsimonious flux balance analysis (pFBA) and flux variability analysis (FVA), were used to interrogate the metabolic properties and capabilities of the reconstructed GSMM for *A. succinogenes* 130Z under varying environmental conditions. Phenotype simulations were performed by maximizing the biomass reaction assuming growth under defined conditions, i.e. defined minimum media containing basic components required for biomass synthesis, such as vitamins, minerals and trace metals and explicit carbon, nitrogen and sulphur sources. Maximum theoretical product yields were calculated by maximizing the target product instead, ignoring the formation of biomass and ATP maintenance requirements, such that the costs of product biosynthesis in terms of carbon, energy and reducing equivalents were properly evaluated.

## Results

### *A. succinogenes* genome-scale metabolic network

The construction of the GSMM for *A. succinogenes* 130Z was carried out in three different phases: (1) first, metabolic functions were assigned to genes; (2) then, biochemical reactions and enzymatic complexes (assigning proper gene-reaction relationships) were compiled to build a draft metabolic model, (3) which was thereafter completed and corrected by defining a biomass reaction, identifying network gaps and correcting inconsistencies when comparing with reported information.

The final *i*BP722 model consists of 722 unique genes (open reading frame (ORF) coverage − 35%), 1072 reactions and 713 unique metabolites. The model is available as a Systems Biology Markup Language (SBML) file at http://darwin.di.uminho.pt/models and BioModels database [61] assigned with the identifier MODEL1804130001 and detailed information on the curated metabolic network can be found in Additional file 2.

### Model validation

#### Predicted growth on different carbon sources

The *i*BP722 model was inspected for the ability to simulate the *A. succinogenes* growth on different conditions. Model simulations were performed using the OptFlux software [62] applying FBA-based methods that maximize the biomass reaction under defined conditions, i.e. exchange fluxes were constrained to specific values (usually experimentally measured fluxes) that allow testing growth under defined environmental conditions, such as sole carbon sources. Some exchange fluxes like those associated with the exchange of CO_2_, NH_4_^+^, Pi, H^+^, vitamins and trace elements were kept unconstrained (i.e. unlimited uptake rates) to provide unlimited basic nutrients for biomass synthesis.

*A. succinogenes* is auxotrophic for three amino acids: L-glutamate, L-cysteine and L-methionine. Glutamate auxotrophy is due to the organism’s inability to synthesize α-ketoglutarate, since the genes encoding for isocitrate dehydrogenase and α-ketoglutarate oxidoreductase enzymes are absent in the genome. *A. succinogenes* possesses most of the genes encoding enzymes associated with the cysteine biosynthetic pathway, but the absence of an adenylsulfate kinase to assimilate sulfate prevents the synthesis of hydrogen sulfide required for the synthesis of L-cysteine. This organism also lacks several genes for the biosynthesis of methionine as identified during the functional annotation process. Given that, exchange fluxes associated with amino acid auxotrophies in *A. succinogenes,* such as L-cysteine and L-methionine, were maintained unconstrained, except for L-glutamate that was limited to a minimum flux value to support growth requirements and avoid glutamate consumption as an additional carbon source. Further flux constraints were introduced when inspecting carbon flux distributions in the central carbon metabolism, and are further detailed in Table SI 11 (Additional file 2). For instance, succinyl-CoA ligase reaction was limited to a zero flux to avoid the formation of succinic acid from succinyl-CoA, in order to be consistent with in vivo observations [33]. Assuming these flux constraints, growth predictions were computed and compared with in vivo observations [4, 27].

Growth phenotypes on 22 different carbon sources under anaerobic conditions were tested and compared with in vivo growth data from [50] (Table 2 and Additional file 3).

**Table 2:** Comparison of growth predictions and in vivo tests on 22 carbon sources

		In vivo	
		Growth	No growth
*In silico*	Growth	TP = 19 (86%)	FP = 0 (0%)
	No growth	FN = 2 (9%)	TN = 1 (5%)

True Positives (TP) and True Negatives (TN) indicate the number of carbon sources in which growth phenotypes were correctly predicted, while False Positives (FP) and False Negatives (FN) indicate the number carbon sources in which *in silico* predictions did not match in vivo observations

The model predicted accurately growth on more than 90% of the carbon sources, with only two not supporting *in silico* growth (FN = 2). *In silico* growth on beta-gentiobiose and D-arabitol was not predicted, as catabolic and transport reactions were not identified in *A. succinogenes*. Further information on transport activities included in the model is given in Additional file 4.

#### Predicted yields for fermentation products

**Fig. 2:**
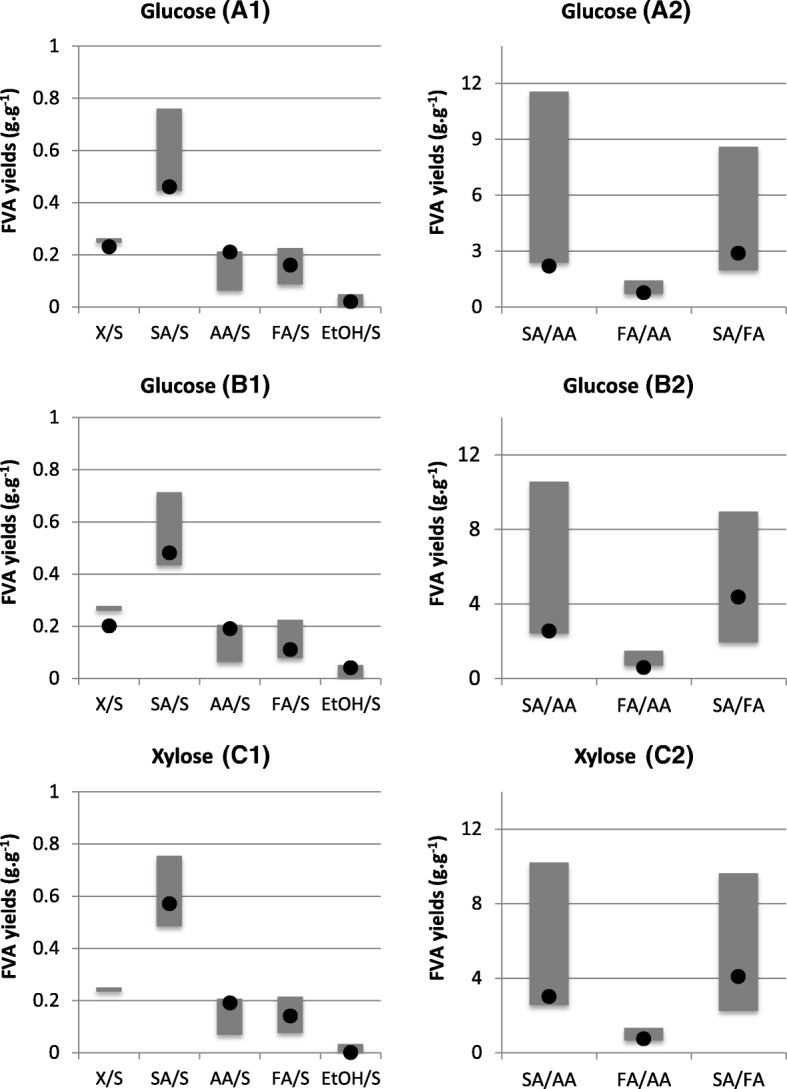
Experimental values (filled dots) versus predicted FVA yields (floating bars) from *A. succinogenes* 130Z cultures on glucose (A and B) and xylose (C). Predicted minimum and maximum FVA yields for biomass, SA, AA, FA and EtOH (Y_x/S,_ Y_SA/S,_ Y_AA/S_, Y_FA/S_, Y_EtOH/S_, respectively) were estimated while maintaining 95% of the maximum biomass formation (A1, B1 and C1). Similarly, minimum and maximum FVA yields between fermentative by-products (SA/AA, FA/AA and SA/FA) were estimated while maintaining 95% of the maximum biomass formation (A2, B2 and C2). *In silico* predictions were performed by setting the substrate uptake rate (*q*_*S*_) to the experimental value (except in condition C that was set to 8 mmol.gDCW^− 1^.h^− 1^). Experimental parameters for conditions A and B were obtained from batch cultures with defined medium (AM3) supplemented with 50 mM glucose and 150 mM NaHCO_3_ under anaerobic conditions [32, 51]; while parameters for C were obtained from a continuous culture at a dilution rate of 0.05 h^− 1^ under anaerobic conditions with supplemented medium (6 g.L^− 1^ yeast extract, 10 g.L^− 1^ corn steep liquor and 50–85 g.L^− 1^ xylose). No biomass yield was experimentally determined for this condition [63]. Mass yields are given in g.g^− 1^

The *i*BP722 model was further validated by predicting anaerobic production yields and comparing with experimental data from batch and/or chemostat cultures of *A. succinogenes* 130Z growing on glucose or xylose at different initial concentrations. [32, 51, 63] (Fig. 2). For each condition, carbon uptake rates were defined based on experimental values, except for condition C that was set to 8 mmol.gDCW^− 1^.h^− 1^, and then predicted production rates were used to calculate minimum and maximum FVA yields for biomass, SA, AA, FA and EtOH (Yx/S, YSA/S, YAA/S, YFA/S, YEtOH/S, respectively). FVA spans are given by the difference between the maximum and minimum predicted yields while maintaining 95% of the maximum biomass formation.

FVA predictions indicate higher yields for C4 by-products (i.e. SA) compared to C3 by-products (i.e. AA, EtOH and FA), which is in good agreement with experimental data; however predicted mass ratios between fermentative by-products, specifically SA/AA and SA/FA, are higher compared with in vivo observations, especially when considering maximum FVA ratios. Yet, FVA spans indicate a significant flexibility for these ratios, which might explain variations in the in vivo observations.

#### Improving model predictions

**Fig. 3:**
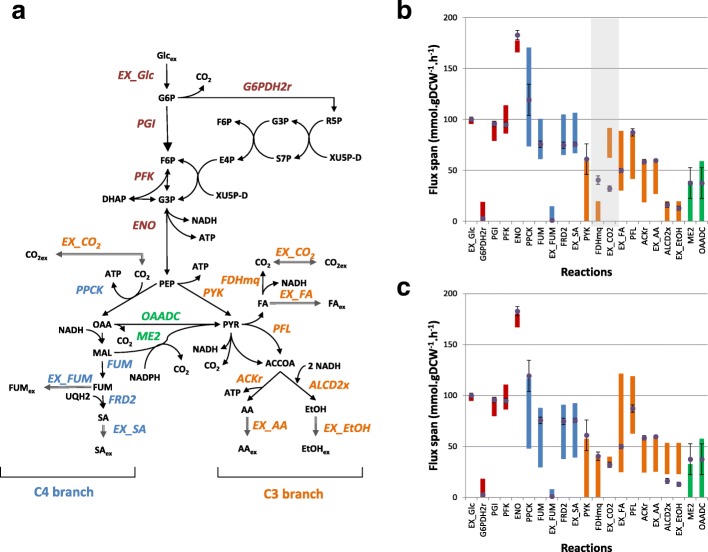
Metabolic flux spans of the *A. succinogenes* central carbon metabolism. The FVA analysis covered individual reactions represented in (a). Flux spans (represented by floating coloured bars) define the flux range of individual reactions while maintaining 95% of the maximum biomass formation (b) and further constraining the maximum uptake rate of CO_2_ to 4 mmol.gDCW^− 1^.h^− 1^ (c). In vivo flux measurements from ^13^C-labeling experiments [33] (represented by filled dots with error bars) are also depicted.

An FVA analysis was performed to elucidate these discrepancies and the metabolic flexibility associated with C3 and C4 metabolic pathways in *A. succinogenes* was examined. Given a set of flux constraints (e.g. substrate uptake rates, q_*S*_), the predicted flux spans for the main reactions in the central carbon metabolism were calculated (Fig. 3a). Flux spans are given by the difference between the maximum and minimum predicted flux values of each metabolic reaction while maintaining 95% of the maximum biomass formation. As shown in Fig. 3b, the largest flux spans were associated with reactions around the PEP node (e.g. PEP carboxykinase (PPCK) and pyruvate kinase (PYK)), while reactions associated to the Embden-Meyerhoff-Parnas pathway (glucose-6-phosphate isomerase (PGI), phosphofructokinase (PFK) and enolase (ENO)) presented the lowest flux spans. Metabolic flux data estimated from ^13^C-labeling experiments [33] was further used to assess the accuracy of predicted flux spans. Most in vivo measurements were between estimated flux ranges, except for the CO_2_ exchange and formate dehydrogenase (FDHmq) reactions, indicating that the NADH-producing FDHmq reaction should be active and CO_2_ exchange flux should be lower. Changes in flux constraints associated with CO_2_ uptake (from unlimited to a maximum of 4 mmol.gDCW^− 1^.h^− 1^) altered predicted flux spans (Fig. 3c), especially for FDHmq, pyruvate-formate lyase (PFL) and the FA exchange reaction (EX_FA), indicating a higher flexibility in metabolic activities linked to FA accumulation.

**Fig. 4:**
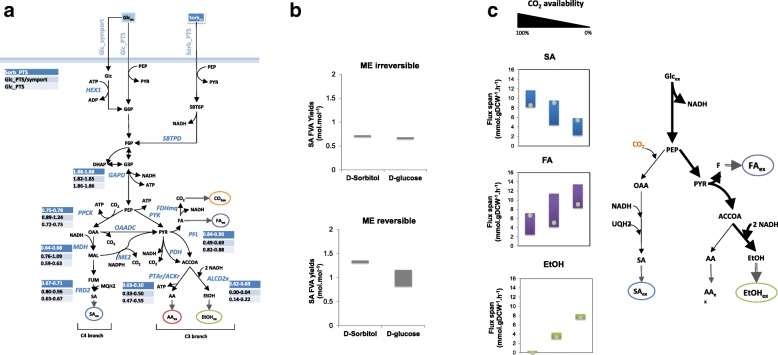
FVA analysis of *A. succinogenes* metabolism under varying growth conditions. Minimum and maximum FVA yields were computed maintaining 99% of the maximum growth rate and are given in mol.mol^− 1^. (a) Predicted FVA yields (minimum-maximum) for key metabolic reactions under D-sorbitol growth conditions (considering PEP:PTS transport mechanism, Sorb_PTS) and glucose conditions (considering both PEP:PTS and symport transport mechanisms, Glc_PTS/symport, or only PEP:PTS transport, Glc_PTS). (b) Predicted FVA yields for SA production when changing ME2 reversibility, both under D-sorbitol or D-glucose conditions (considering only PEP:PTS transport). (c) Predicted FVA yields and pFBA yields (grey dots) for SA, FA and EtOH production under D-glucose conditions when changing CO_2_ availability (100% availability means unconstrained CO_2_ uptake, while 0% indicates a zero CO_2_ uptake flux value)

The production of SA in *A. succinogenes* is influenced by several factors, namely the utilized carbon sources [64] or the availability of CO_2_ [5, 33, 48]. The *i*BP722 model was investigated for predicting the metabolic flexibility associated with the production of SA when changing CO_2_ availability or carbon sources, as well as the reversibility of metabolic reactions like malic enzyme (ME2) (Fig. 4). FVA yields for maximum growth simulations show that the production of reduced by-products, particularly EtOH, changes with the carbon source. As presented in Fig. 4a, the minimum and maximum FVA yields for ALCD2x under D-sorbitol growth conditions (0.62 and 0.69 mol.mol^− 1^) were higher compared to glucose (0.14 and 0.22 mol.mol^− 1^, respectively), with the consequent accumulation of higher amounts of EtOH. Under glucose conditions most of the carbon flux through the C3 branch is redirected toward the production of AA instead, via acetate kinase (ACKr) with the production of ATP (minimum and maximum FVA yields of 0.47 and 0.55 mol.mol^− 1^, respectively). Interestingly, however, is that predicted FVA yields for SA production hardly change between glucose and D-sorbitol growth conditions when considering the same transport mechanism, i.e. PEP:sugar phosphotransferase system (PEP:PTS). Yet, assuming that both transport mechanisms could be active (i.e., symport and PEP:PTS) for glucose uptake would increase SA minimum and maximum FVA yields from 0.63 and 0.67 to 0.82 and 0.96 mol.mol^− 1^, respectively.

The flux exchange between the C3 and C4 branches has been also investigated as a major factor affecting the metabolic flexibility of sugars fermentation in *A. succinogenes* [33]. The decarboxylation of L-malate to pyruvate (PYR) reducing NADP^+^ to NADPH by ME2, may have a major role in the fermentative metabolism. Although the thermodynamics of this reaction is not conclusive regarding its reversibility, the metabolic network was tested using both the forward and the reverse directions. FVA results (Fig. 4b) show that SA production should increase when ME2 occurs in the reverse direction, especially for D-sorbitol conditions, redirecting most of the PYR pool toward the C4 branch. Consequently, a greater metabolic flexibility in SA production, especially under glucose growth conditions, was predicted.

The availability of CO_2_ was also shown to influence the production of SA (Fig. 4c). Model predictions indicated that maximum SA yields on glucose can decrease nearly 20% when decreasing the maximum CO_2_ uptake rates by 50%. A shift in carbon flux distributions is observed when decreasing CO_2_ uptake levels, redirecting most of the carbon flux toward the production of C3 by-products like FA and EtOH.

## Discussion

GSMMs are powerful tools to explore the metabolism of biological systems. In this work, the *i*BP722 model of *A. succinogenes* 130Z was reconstructed and used as a platform for the *in silico* analysis of the metabolic behaviour of this organism during anaerobic growth. The major end-product is SA, but significant amounts of other by-products such as EtOH, FA and AA are also accumulated. The possibility to predict and adjust the fermentative metabolism of *A. succinogenes* 130Z under different conditions brings new opportunities to exploit this host as a platform for the industrial production of SA and other reduced by-products.

*In silico* simulations were carried out using pFBA and FVA methods predicting growth behaviour under chemically defined medium. Model predictions were validated using reported physiological data and flux distributions from ^13^C experiments found in literature [32, 33, 51]. The *i*BP722 model supports growth predictions on 19 carbon sources under anaerobic conditions (Additional file 3), including C6 and C5 sugars that were found to enter the cell mainly through active transport systems (Additional file 4). The only two carbon sources with incorrect growth predictions were β-gentiobiose and D-arabitol, due to the absence of transport and catabolic pathways in the model, since no coding proteins were found. On the other hand, no-growth predictions for glycerol were correctly identified, though transport and catabolic reactions for glycerol consumption are present in the model. According to in vivo growth experiments [65], no cellular growth on glycerol as a sole carbon source is observed under anaerobic conditions, possibly caused by redox imbalance under anaerobiosis. However, the addition of external electron acceptors like dimethylsulfoxide (DMSO) has shown to recover cellular growth [35], which was also confirmed by *in silico* analysis (Additional file 5).

Further validations included the comparison of in vivo measurements and *in silico* FVA predictions for minimum and maximum yields under various conditions (Fig. 2). Despite being relatively variable (from 0.20 to 0.23 g.g^− 1^ under glucose conditions), experimental values for biomass yields were used to validate model predictions. Variations in experimental conditions, particularly associated with culture media that is often supplemented with yeast extract, interfering with carbon yields, or the initial sugar concentration that may affect bacterial growth due to substrate inhibition [66], may explain these differences. Nevertheless, biomass composition represented in the model is based on the work of McKinlay and co-workers [51], which is expected to provide accurate *in silico* predictions, especially regarding metabolic requirements to generate biomass contents *per* unit of substrate (i.e. biomass yields, Y_x/s_). Additional reaction constraints were included in the model to improve model predictions. For instance, flux constraints of L-glutamate uptake and succinyl-CoA synthase were changed to improve carbon-to-nitrogen ratios according to experimental measurements [51]. Moreover, the energetic requirement for non-growth associated maintenance, i.e. the amount of ATP spent for cellular maintenance without growth, was adjusted to improve predicted biomass yields.

Fermentative products ratios were also compared, showing some consistency between experimental measurements and FVA predictions. Predicted mass ratios for SA/AA and SA/FA are higher than for SA/FA, which is consistent with in vivo data; but FVA spans for SA/AA and SA/FA suggest a huge flexibility in these ratios. This suggests that carbon flux distributions in the C3 branch are rather challenging to predict. In fact, FVA flux spans of fermentative pathways (Fig. 3b) indicate a high metabolic flexibility in the accumulation of by-products which, in some cases are inconsistent with experimental measurements. However, when using data from ^13^C-labeling experiments [51] to constrain fluxes, in particular the CO_2_ uptake rate (Fig. 3c), model predictions improved, especially for reactions associated with FA accumulation (PFL, FDHmq and EX_FA).

Overall, the *i*BP722 model allow us to evaluate the production of SA, showing that maximum theoretical yield for SA (1.1 g *per* g of glucose, assuming a symport system and no ATP requirements for maintenance) is comparable to those predicted using *E. coli* or *S. cerevisiae* models (1.1 and 0.8 g *per* g of glucose for anaerobic conditions using iJO1366 [67] and iMM904 [68], respectively, under the same previous assumptions). Thus, metabolic capabilities seem equivalent to other organisms being exploited for SA production. It also allows describing the impact of growth conditions on the production of C3 and C4 fermentative by-products. The carbon split between C4 and C3 pathways has been investigated and showed to be influenced by various factors like the available reducing power (i.e. NADH/NAD^+^ ratio) or CO_2_ availability, therefore affecting the SA production in *A. succinogenes* growing cultures [64, 69]. *In silico* predicted SA yields are higher with more reduced carbon sources (e.g. minimum and maximum FVA yields of 0.44 and 0.46 g.g^− 1^ on sorbitol compared to 0.41 and 0.44 g.g^− 1^ on glucose, correspondingly, assuming the same sugar transport mechanism) and higher CO_2_ availability, favouring carbon flux through the C4 branch, as a consequence of higher reducing power and higher PPCK carboxylation activity. Moreover, flexibility in sugar transport mechanisms or enzymes reversibility (e.g. ME2) may lead to increased levels of SA, as carbon flux partitioning between C3 and C4 pathways was shown to be largely affected (Fig. 4). The reverse activity of ME2 has shown to increase SA yields, redirecting part of the carbon flux from the C3 toward the C4 branch through the carboxylation of pyruvate to L-malate, with the simultaneous production of reducing power (NADPH). On the other hand, transport activities limited to PEP:PTS-based systems decrease SA yields, since PEP is used as the energy source for sugar uptake generating pyruvate, which is necessarily consumed via the C3 branch.

## Conclusions

In this work, the GSMM of *A. succinogenes* 130Z (*i*BP722) was reconstructed and validated using different sets of experimental data from literature. The reconstruction of the model included the compilation of functionally annotated metabolic genes and the corresponding coding proteins, as well as associated biochemical reactions. The model was complemented with a biomass equation and spontaneous and transport reactions. It was further amended after a gap filling process, including the identification of genetic evidences based on homology searches. Model accuracy to predict growth phenotypes and the production of fermentative by-products was evaluated using FVA and pFBA simulation methods. The ability to predict changes in carbon flux distributions due to environmental perturbations like CO_2_ limitations or alterations in the redox state was also tested. Predicted SA yields were in good agreement with experimental data, suggesting that the model is able to characterize the fermentative metabolism under various conditions. The increase in CO_2_ availability showed to have a positive impact in SA yields, which is consistent with reported data [48, 49]. As such, optimal conditions for increased SA yields may include increased CO_2_ availability, the use of more reduced carbon sources like sorbitol or the use of external energy source like H_2_ [33]. Besides improving process conditions for the production of SA, the design of microbial strains by metabolic engineering to increase the flux through the C4 branch has been attempted, albeit with limited success [70]. Model predictions show that changes in sugar transport systems, CO_2_ fixation activities or the reversibility of the malic enzyme may have an impact in the SA yield. Therefore, it is expected that modifications in the metabolism that would include these activities would further improve the SA yield.

Overall, the *i*BP722 model enables a better understanding of the metabolic behaviour and capabilities of this organism, which can be explored to further improve SA productivity. The capacity to consume a wide range of C5 and C6 sugars, as well as other low-cost carbon sources (e.g. glycerol or lactose) and its metabolic flexibility may provide some advantages over other SA-producing strains, like recombinant *Escherichia coli* or *Mannheimia succiniciproducens* [69]*.*

## Additional files

Additional file 1: Biomass composition of *A. succinogenes* 130Z. (DOCX 43 kb)

Additional file 2: Details on the *i*BP722 model. (DOCX 24 kb)

Additional file 3: Testing *A. succinogenes* growth on different carbon sources. (DOCX 22 kb)

Additional file 4: Details on transport mechanisms included in the *i*BP722 model. (DOCX 37 kb)

Additional file 5: Growth predictions on glycerol with and without DMSO. (DOCX 21 kb)

## Metabolites

AA: Acetic acid
AA_ex_: Extracellular acetic acid
ACCOA: Acetyl-coenzyme A
E4P: Erythrose-4-phosphate
EtOH: Ethanol
EtOH_ex_: Extracellular ethanol
F6P: Fructose-6-phosphate
FA: Formic acid
FA_ex_: Extracellular formic acid
FUM: Fumarate
FUM_ex_: Extracellular fumarate
G3P: Glyceraldehyde-3-phosphate
G6P: Glucose-6-phosphate
Glc_ex_: Extracellular glucose
MAL: Malate
OAA: Oxaloacetate
PEP: Phosphoenolpyruvate
PYR: Pyruvate
R5P: Ribose-5-phosphate
S7P: Sedoheptulose-7-phosphate
SA: Succinic acid.
SA_ex_: Extracellular succinic acid

## Reactions

ACKr: Acetate kinase
ALCD2x: Alcohol dehydrogenase
ENO: Enolase
EX_CO_2_: Carbon dioxide exchange reaction
EX_AA: Acetic acid exchange reaction
EX_EtOH: Ethanol exchange reaction
EX_FA: Formic acid exchange reaction
EX_FUM: Fumarate exchange reaction
EX_Glc: Glucose exchange reaction
EX_SA: Succinic acid exchange reaction
FDHmq: Formate dehydrogenase
FRD2: Fumarate reductase
FUM: Fumarase
G6PDH2r: Glucose 6-phosphate dehydrogenase of the oxidative pentose phosphate pathway
ME2: Malic enzyme
OAADC: Oxaloacetate decarboxylase
PFK: Phosphofructokinase
PFL: Pyruvate formate-lyase
PGI: Glucose-6-phosphate isomerase
PPCK: PEP carboxykinase
PYK: Pyruvate kinase
