# Supplementary material for: Reconstruction of a genome-scale metabolic model for Actinobacillus succinogenes 130Z
Source: BMC Syst Biol. 2018 May 30;12:61. doi: 10.1186/s12918-018-0585-7 (PMC5975692; doi:10.1186/s12918-018-0585-7)
Supplement: Supplementary file 1 — Biomass composition of A. succinogenes 130Z. (DOCX 43 kb) [file 12918_2018_585_MOESM1_ESM.docx]

Additional file 1: Biomass composition of *A. succinogenes* 130Z

- **Overall Composition**

The overall cellular composition was obtained from literature sources ^1^ and was assumed to have eight essential macromolecular components: proteins, DNA, RNA, phospholipids, peptidoglycan, lipopolysaccharide (LPS), glycogen and the small molecule pool that include cofactors and vitamins (CAV).

Table SI 1. Biomass composition of *A. succinogenes* 130Z.

| **Macromolecule** | **% of dry cell weight** |
| --- | --- |
| Protein | 56.8 |
| DNA | 4.4 |
| RNA | 14.2 |
| Lipopolysaccharide (LPS) | 4.7 |
| Phospholipids | 8.8 |
| Peptidoglycan | 3.5 |
| Glycogen | 6.6 |
| Cofactors and Vitamins (CAV) | 1.0 |
| **Total** | **100.0** |

- **Protein Composition**

The average amino acids composition of proteins was calculated based on the *A. succinogenes* 130Z genome according to the methodology proposed in ^2^.

Table SI 2. Average amino acids composition

| **Metabolite ID** | **Metabolite name** | **mmol *per* gram of protein** |
| --- | --- | --- |
| ala-L | L-Alanine | 1.48319 |
| arg-L | L-Arginine | 0.28129 |
| asn-L | L-Asparagine | 0.43119 |
| asp-L | L-Aspartate | 0.42752 |
| cys-L | L-Cysteine | 0.15327 |
| glu-L | L-Glutamate | 0.40834 |
| gln-L | L-Glutamine | 0.41146 |
| gly | Glycine | 1.78648 |
| his-L | L-Histidine | 0.14094 |
| ile-L | L-Isoleucine | 0.48144 |
| leu-L | L-Leucine | 0.76082 |
| lys-L | L-Lysine | 0.53462 |
| met-L | L-Methionine | 0.14733 |
| phe-L | L-Phenylalanine | 0.27463 |
| pro-L | L-Proline | 0.41617 |
| ser-L | L-Serine | 0.44393 |
| thr-L | L-Threonine | 0.50404 |
| trp-L | L-Tryptophan | 0.05663 |
| tyr-L | L-Tyrosine | 0.15082 |
| val-L | L-Valine | 0.76211 |

- **DNA Composition**

The average DNA composition was calculated based on the *A. succinogenes* 130Z genome according to the methodology proposed in ^2^.

Table SI 3. Average DNA composition

| **Metabolite ID** | **Metabolite name** | **mmol *per* gram DNA** |
| --- | --- | --- |
| datp | dATP | 0.79553 |
| dttp | dTTP | 0.79553 |
| dgtp | dGTP | 0.82800 |
| dctp | dCTP | 0.82800 |

- **RNA Composition**

The average RNA composition was calculated based on the *A. succinogenes* 130Z genome according to the methodology proposed in ^2^.

Table SI 4. Average RNA composition

| **Metabolite ID** | **Metabolite name** | **mmol *per* gram RNA** |
| --- | --- | --- |
| atp | ATP | 7.4521 |
| ctp | CTP | 0.60859 |
| gtp | GTP | 0.88804 |
| utp | UTP | 0.81352 |

- **Peptidoglycan composition**

The average peptidoglycan composition was assumed to be identical to *E. coli* ^3^.

Table SI 5. Average peptidoglycan composition

| **Metabolite ID** | **Metabolite name** | **mmol *per* gram peptidoglycan** |
| --- | --- | --- |
| udcpdp | di-*trans*,poly-*cis*-Undecaprenyl diphosphate | 0.00220 |
| murein3p3p | two linked disaccharide tripeptide murein units (uncrosslinked, middle of chain) | 0.05368 |
| murein4px4px4p | three disaccharide linked murein units (tetrapeptide crosslinked tetrapeptide (A2pm->D-ala) & tetrapeptide crosslinked tetrapeptide (A2pm->D-ala)) (middle of chain) | 0.02686 |
| murein4p4p | two linked disaccharide tetrapeptide murein units (uncrosslinked, middle of chain) | 0.21477 |
| murein3px4p | two disaccharide linked murein units, tripeptide crosslinked tetrapeptide (A2pm->D-ala) (middle of chain) | 0.02415 |
| murein4px4p | two disaccharide linked murein units, tetrapeptide crosslinked tetrapeptide (A2pm->D-ala) (middle of chain) | 0.21745 |

- **Phospholipid composition**

The phospholipid content was assumed to be identical to *E. coli* ^3^. Phospholipid composition was then assumed to be 25% (w/w) phosphatidylglycerol and 75% (w/w) phosphatidylethanolamine.

Table SI 6. Average phospholipid composition

| **Metabolite ID** | **Metabolite name** | **mmol *per* gram phospholipid** |
| --- | --- | --- |
| phptglyc | Phosphatidylglycerol | 0.34305 |
| pe | Phosphatidylethanolamine | 1.07489 |

- **Small molecules or cofactors and vitamins (CAV) pool composition**

For the CAV composition, the selected molecules were assumed to be the same as for *E. coli* ^3^, with minor adjustments in biomass contents. As most of these small molecules are considered “universal” and present in most bacteria^4^; each small molecule with putative encoding-genes for its biosynthesis in *A. succinogenes* genome was therefore included in CAV composition.

Table SI 7. Average CAV pool composition

| **Metabolite ID** | **Metabolite name** | **mmol *per* gram CAV** |
| --- | --- | --- |
| ptrc | Putrescine | 1.9058 |
| nadp | Nicotinamide adenine dinucleotide phosphate | 0.0064 |
| nad | Nicotinamide adenine dinucleotide | 0.1024 |
| 10fthf | 10-Formyltetrahydrofolate | 0.0128 |
| mlthf | 5,10-Methylenetetrahydrofolate | 0.0128 |
| 5mthf | 5-Methyltetrahydrofolate | 0.0128 |
| succoa | Succinyl-CoA | 0.0056 |
| gthrd | Glutathione | 0.0128 |
| malcoa | Malonyl-CoA | 0.0018 |
| accoa | Acetyl-Coenzyme A | 0.0160 |
| 2fe2s | Fe2S2 iron-sulfur cluster | 0.0014 |
| molybenz_molyb_cof | Molybdoenzyme molybdenum cofactor | 0.0066 |
| amet | S-Adenosyl-L-methionine | 0.0128 |
| thf | Tetrahydrofolate | 0.0128 |
| fad | Flavin adenine dinucleotide | 0.0128 |
| adocbl | Adenosylcobalamin | 0.0128 |
| spmd | Spermidine | 0.3863 |
| btn | Biotin | 0.0001 |
| pheme | Heme b | 0.0128 |
| ribflv | Riboflavin | 0.0128 |
| mqn8 | Menaquinone-8 | 0.0128 |
| sheme | Siroheme | 0.0128 |
| hemeO | Heme O | 0.0128 |
| chor | Chorismate | 0.0128 |
| thmpp | Thiamin diphosphate | 0.0128 |
| pydx5p | Pyridoxal phosphate | 0.0128 |
| 4fe4s | 4Fe-4S iron-sulfur center | 0.0142 |
| coa | Coenzyme A | 0.0096 |
| 2dmmql8 | Demethylmenaquinol-8 | 0.0128 |
| nh4 | Ammonium | 0.7090 |
| cu2 | Copper | 0.0386 |
| mn2 | Manganese | 0.0377 |
| fe3 | Ferric iron | 0.4256 |
| ca2 | Calcium | 0.2837 |
| cobalt2 | Cobalt | 0.0014 |
| so4 | Sulfate | 0.2364 |
| mg2 | Magnesium | 0.4727 |
| ni2 | Nickel | 0.0176 |
| cl | Chloride | 0.2836 |
| zn2 | Zinc | 0.0186 |
| fe2 | Ferrous iron | 0.3660 |
| k | Potassium | 10.6370 |

- **References**

1. McKinlay JB, Shachar-Hill Y, Zeikus JG, Vieille C. Determining *Actinobacillus succinogenes* metabolic pathways and fluxes by NMR and GC-MS analyses of 13C-labeled metabolic product isotopomers. *Metab Eng*. 2007;9(2):177-192. doi:10.1016/j.ymben.2006.10.006.

2. McKinlay JB, Laivenieks M, Schindler BD, et al. A genomic perspective on the potential of *Actinobacillus succinogenes* for industrial succinate production. *BMC Genomics*. 2010;11(1):680. doi:10.1186/1471-2164-11-680.

3. Orth JD, Conrad TM, Na J, et al. A comprehensive genome-scale reconstruction of *Escherichia coli* metabolism--2011. *Mol Syst Biol*. 2014;7(1):535-535. doi:10.1038/msb.2011.65.

4. Xavier JC, Patil KR, Rocha I. Integration of Biomass Formulations of Genome-Scale Metabolic Models with Experimental Data Reveals Universally Essential Cofactors in Prokaryotes. *Metab Eng*. 2017;39:200-208. doi:10.1016/j.ymben.2016.12.002.
