# Supplementary material for: Reconstruction of a genome-scale metabolic model for Actinobacillus succinogenes 130Z
Source: BMC Syst Biol. 2018 May 30;12:61. doi: 10.1186/s12918-018-0585-7 (PMC5975692; doi:10.1186/s12918-018-0585-7)
Supplement: Supplementary file 2 — Details on the iBP722 model. (DOCX 24 kb) [file 12918_2018_585_MOESM2_ESM.docx]

Additional file 2: Details on *i*BP722 model.

Table SI 8. Statistics on the metabolic functional annotation of *A. succinogenes* ORFs

| Total number of ORFs  Number of validated reactions | 2079  603 |
| --- | --- |
| Number of genes with enzymatic function | 517 |
| Number of genes with transporter function | 168 |
| Total number of genes with enzymatic or transporter function | 730 |
| Number of validated EC numbers  Number of Metabolites | 439  664 |

Table SI 9. Statistics on the *i*BP722 model

| Total number of reactions  Number of enzymatic reactions  Number of transporter reactions  Drains | 1072  828  117  127 |
| --- | --- |
| Genes | 722 |
| Metabolites | 713 |

Table SI 10. Metabolic reactions included in the gap filling process.

| **Reaction ID** | **Stoichiometry** | **Associated genes** | **Associated enzyme** |
| --- | --- | --- | --- |
| MTHFR2 | H+ [c] + 5,10-Methylenetetrahydrofolate [c] + NADH [c] ==> 5-Methyltetrahydrofolate [c] + NAD+ [c] | Unknown | 5,10-methylenetetrahydrofolate reductase |
| ASPCT | L-Aspartate [c] + Carbamoyl phosphate [c] ==> N-Carbamoyl-L-aspartate [c] + Orthophosphate [c] | Unknown | Aspartate carbamoyltransferase |
| AMDC | S-Adenosyl-L-methionine [c] + H+ [c] ==> S-Adenosylmethioninamine [c] + CO2 [c] | Unknown | adenosylmethionine decarboxylase |
| AMMQLT8 | 2-Demethylmenaquinone [c] + S-Adenosyl-L-methionine [c] ==> S-Adenosyl-L-homocysteine[c] + Menaquinone [c] | Asuc_0007 | Demethylmenaquinone methyltransferase |

**Table SI 11. Lower and upper bounds used to perform model simulations.** To note that for each carbon source condition, the corresponding bounds need to be altered.

| **Reactions** | **[Min, Max] Flux constraints (mmol.gCDW^-1^.h^-1^)** |
| --- | --- |
| Maintenance ATP (ATPM) | [4, 1000] |
| Glutamate exchange (EX_glu_L_)  ALCD19  NAR  MDH  SUCR  GLUDy  AKGDHE1  PSERT  MALT  GLCtpc  GND  G6PDH2r  HEX1  GLYCDHmq  SUCOAS  FRD3 | [-4.2, 1000]  [0, 0]  [0, 0]  [-1000, 0]  [0, 1000]  [0, 1000]  [0, 1000]  [-1000, 0]  [0, 1000]  [0, 1000]  [0, 1000]  [0, 1000]  [0, 1000]  [0, 0]  [0, 0]  [0, 0] |
