# Supplementary material for: Reconstruction of a genome-scale metabolic model for Actinobacillus succinogenes 130Z
Source: BMC Syst Biol. 2018 May 30;12:61. doi: 10.1186/s12918-018-0585-7 (PMC5975692; doi:10.1186/s12918-018-0585-7)
Supplement: Supplementary file 3 — Testing A. succinogenes growth on different carbon sources. (DOCX 22 kb) [file 12918_2018_585_MOESM3_ESM.docx]

Additional file 3: Testing *A. succinogenes* growth on different carbon sources

Table SI 12. Comparison between *in vivo* observations [4, 46] and model predictions for growth under different carbon sources. (+) growth; (-) no growth

| **Carbon source** | ***In vivo*** | ***In silico*** |
| --- | --- | --- |
| D-Glucose | + | + |
| D-Fructose | + | + |
| D-Xylose | + | + |
| Maltose | + | + |
| Lactose | + | + |
| Galactose | + | + |
| Gluconate | + | + |
| Sorbitol | + | + |
| Mannose | + | + |
| D-Mannitol | + | + |
| Sucrose | + | + |
| 5-Dehydro-D-Gluconate | + | + |
| L-Arabinose | + | + |
| Glycerol | - | - |
| β-Gentiobiose | + | - |
| D-Arabitol | + | - |
| L-Idonate | + | + |
| Ascorbate | + | + |
| D-Ribose | + | + |
| D-Glucarate | + | + |
| D-Galactarate | + | + |
| Cellobiose | + | + |
