# Supplementary material for: Reconstruction of a genome-scale metabolic model for Actinobacillus succinogenes 130Z
Source: BMC Syst Biol. 2018 May 30;12:61. doi: 10.1186/s12918-018-0585-7 (PMC5975692; doi:10.1186/s12918-018-0585-7)
Supplement: Supplementary file 4 — Details on transport mechanisms included in the iBP722 model. (DOCX 37 kb) [file 12918_2018_585_MOESM4_ESM.docx]

Additional file 4: Details on transport mechanisms included in the *i*BP722 model

Table SI 13. List of transport reactions included in the *i*BP722 model.

| **Transported metabolite** | **Reaction ID** | **Stoichiometry** | **Type of transporter (TC class)** | **Genes** |
| --- | --- | --- | --- | --- |
| **Passive Transport** | | | | |
| Water | H2Ot | H_2_O [p] <-> H_2_O [c] | Diffusion |  |
| Hydrogen | H2fpc | Hydrogen [p] <-> Hydrogen [c] | Diffusion |  |
| Carbon Dioxide | CO2f | CO_2_ [p] <-> CO_2_ [c] | Diffusion |  |
| Acetate | Acf | Acetate [c] <-> Acetate [p] | Permease (2.A.7.3.-) | Asuc_0919 |
| **Active transport** | | | | |
| **Sugars** | | | | |
| D-Ribose | RibABC | ATP [c] + H_2_O [c] + D-Ribose [p] -> ADP [c] + Pi [c] + D-Ribose [c] | ABC transporter (3.A.1.2.-) | Asuc_0195  Asuc_0196  Asuc_0197  Asuc_0081  Asuc_0082  Asuc_0083  Asuc_0172  Asuc_0173 |
| D-Mannitol | MantlPTS | D-Mannitol [p] + Phosphoenolpyruvate [c] -> D-Mannitol 1-phosphate [c] + Pyruvate [c] | PTS (4.A.2.1.-) | Asuc_0455 |
| D-Mannose | ManPTS | D-Mannose [p] + Phosphoenolpyruvate [c] -> D-Mannose 6-phosphate [c] + Pyruvate [c] | PTS (4.A.6.1.-) | Asuc_0286  Asuc_0287  Asuc_0288  Asuc_0585  Asuc_0586  Asuc_0587  Asuc_0936  Asuc_0937  Asuc_0938 |
| D-Glucose | GlcPTS | D-Glucose [p] + Phosphoenolpyruvate [c] -> D-Glucose 6-phosphate [c] + Pyruvate [c] | PTS (4.A.6.1.-) | Asuc_0994  Asuc_0995  Asuc_0996  Asuc_0127  Asuc_0975 |
| D-Glucose | Glcfcp | D-Glucose [c] -> D-Glucose [p] | Permease (2.A.1.20.-) | Asuc_1424 |
| D-Glucose | GlcSpc | D-Glucose [p] + H^+^ [p] <-> D-Glucose [c] + H^+^ [c] | Symporter (2.A.1.1.-) | Asuc_0496 |
| D-Galactose | GalABC | ATP [c] + D-Galactose [p] + H_2_O [c] -> ADP [c] + D-Galactose [c] + Pi [c] | ABC transporter (3.A.1.2.-) | Asuc_1896  Asuc_1897  Asuc_1898 |
| Cellobiose | bGlucPTS | Cellobiose [p] + Phosphoenolpyruvate [c] -> 6-Phospho-beta-D-glucosyl-(1,4)-D-glucose [c] + Pyruvate [c] | PTS (4.A.1.2.-) | Asuc_0975 |
| L-Arabinose | ArabABC | L-Arabinose [p] + ATP [c] + H_2_O [c] -> ADP [c] + L-Arabinose [c] + Pi[c] | ABC transporter (3.A.1.2.-) | Asuc_0489  Asuc_0490  Asuc_0491 |
| D-Glycerate | GlycRSpc | D-Glycerate [p] + H^+^ [p] -> D-Glycerate [c] + H^+^ [c] | Symporter (2.A.8.1.-) | Unknown |
| D-Sorbitol | SorbPTS | D-Sorbitol [p] + Phosphoenolpyruvate [c] -> Pyruvate [c] + Sorbitol 6-phosphate [c] | PTS (4.A.4.1.-) | Asuc_0434  Asuc_0435  Asuc_0436 |
| Sucrose | SucrPTS | Phosphoenolpyruvate [c] + Sucrose [p] -> Pyruvate [c] + Sucrose 6-phosphate [c] | PTS (4.A.1.2.-) | Asuc_0914 |
| Sucrose | SucrSpc | H^+^ [p] + Sucrose [p] <-> H^+^ [c] + Sucrose [c] | Symporter (2.A.1.5.-) | Asuc_1397 |
| Lactose | LctsSpc | H^+^ [p] + Lactose [p] <-> H^+^ [c] + Lactose [c] | Symporter (2.A.1.5.-) | Asuc_1397 |
| Maltose | MaltABC | ATP [c] + H_2_O [c] + Maltose [p] -> ADP [c] + Maltose [c] + Pi[c] | ABC transporter (3.A.1.1.-) | Asuc_0316  Asuc_0317  Asuc_0318  Asuc_0319  Asuc_0320 |
| D-Fructose | FruABC | ATP [c] + D-Fructose [p] + H_2_O [c] -> ADP [c] + D-Fructose [c] + Pi [c] | ABC transporter (3.A.1.1.-) | Asuc_0081  Asuc_0082  Asuc_0083 |
| D-Fructose | FruPTS | D-Fructose [p] + Phosphoenolpyruvate [c] -> D-Fructose 6-phosphate [c] + Pyruvate [c] | PTS (4.A.6.1.-) | Asuc_0286  Asuc_0288  Asuc_0287  Asuc_0936  Asuc_0937  Asuc_0938 |
| D-Xylose | XylSpc | H^+^ [p] + D-Xylose [p] <-> H^+^ [c] + D-Xylose [c] | Symporter (2.A.1.1.-) | Asuc_0128 |
| D-Xylose | XylABC | ATP [c] + H_2_O [c] + D-Xylose [p] -> ADP [c] + Pi [c] + D-Xylose [c] | ABC transporter (3.A.1.1.-, 3.A.1.2.-) | Asuc_0496  Asuc_0497  Asuc_0498  Asuc_0499  Asuc_0081  Asuc_0082  Asuc_0083  Asuc_0172  Asuc_0173 |
| N-Acetylmuramate | AcmumPTS | N-Acetylmuramate [p] + Phosphoenolpyruvate [c] -> N-Acetylmuramic acid 6-phosphate [c] + Pyruvate [c] | PTS (4.A.1.2.-) | Asuc_0914 |
| **Carboxylic acids and alcohols** | | | | |
| Glycerol | GlycSpc | Glycerol [p] + H^+^ [p] -> Glycerol [c] + H^+^ [c] | Symporter (1.A.8.2.-) | Asuc_1603  Asuc_0408 |
| *sn*-Glycerol 3-phosphate | Glyc3pANTI | *sn*-Glycerol 3-phosphate [p] + Pi [c] <-> *sn*-Glycerol 3-phosphate [c] + Pi [p] | Antiporter (2.A.1.4.-) | Asuc_0593 |
| 5-Dehydro-D-gluconate | 5DGlucSYMP | 5-Dehydro-D-gluconate [p] + H^+^ [p] -> 5-Dehydro-D-gluconate [c] + H^+^ [c] | Symporter (2.A.8.1.-) | Asuc_1157  Asuc_1848  Asuc_0889  Asuc_0119 |
| D-Gluconate | DGlucSpc | D-Gluconic acid [p] + H^+^ [p] -> D-Gluconic acid [c] + H^+^ [c] | Symporter (2.A.8.1.-) | Asuc_1157  Asuc_1848  Asuc_0889  Asuc_0119 |
| L-Idonate | IdonSpc | H^+^ [p] + L-Idonate [p] -> H^+^ [c] + L-Idonate [c] | Symporter (2.A.8.1.-) | Asuc_1157 Asuc_1848 Asuc_0119  Asuc_0889 |
| 4-Aminobutanoate | 4AbutSpc | 4-Aminobutanoate [p] + Cl^-^ [p] -> 4-Aminobutanoate [c] + Cl^-^ [c] | Symporter (2.A.22.3.-) | Asuc_0284 |
| Succinate | SuccSpc | H^+^ [p] + Succinate [p] <-> H^+^ [c] + Succinate [c] | Symporter (2.A.56.1.-) | Asuc_0146  Asuc_0147  Asuc_0148  Asuc_0156  Asuc_0157  Asuc_0158  Asuc_0270  Asuc_0271  Asuc_0272  Asuc_0366  Asuc_0367  Asuc_0368  Asuc_1163  Asuc_1164  Asuc_1165  Asuc_1577  Asuc_1578  Asuc_1579  Asuc_1921  Asuc_1922  Asuc_1923 |
| Succinate | SuccSipc | Sodium [p] + Succinate [p] -> Sodium [c] + Succinate [c] | Symporter (2.A.47.1.-) | Asuc_1568 |
| Citrate/Succinate | SuccCitANTI | Citrate [p] + Succinate [c] -> Citrate [c] + Succinate [p] | Antiporter (2.A.47.3.2) | Asuc_0183 Asuc_1482 |
| Citrate | CitSpc | Citrate [p] + H^+^ [p] -> Citrate [c] + H^+^ [c] | Symporter (2.A.11.1.-) | Asuc_1303 |
| S-Malate | MalSipc | (S)-Malate [p] + Sodium [p] -> (S)-Malate [c] + Sodium [c] | Symporter (2.A.47.1.-) | Asuc_1568 |
| S-Malate | MalSpc | H^+^ [p] + (S)-Malate [p] <-> H^+^ [c] + (S)-Malate [c] | Symporter (2.A.56.1.-) | Asuc_0146  Asuc_0147  Asuc_0148  Asuc_0156  Asuc_0157  Asuc_0158  Asuc_0270  Asuc_0271  Asuc_0272  Asuc_0366  Asuc_0367  Asuc_0368  Asuc_1163  Asuc_1164  Asuc_1165  Asuc_1577  Asuc_1578  Asuc_1579  Asuc_1921  Asuc_1922  Asuc_1923 |
| 2-Oxoglutarate/S-Malate | MalAkgANTI | 2-Oxoglutarate [p] + (S)-Malate [c] -> 2-Oxoglutarate [c] + (S)-Malate [p] | Antiporter (2.A.47.3.-) | Asuc_0183 Asuc_1482 |
| Fumarate/S-Malate | MalFumANTI | Fumarate [p] + (S)-Malate (mal-L)[c] <-> Fumarate (fum)[c] + (S)-Malate (mal-L)[p] | Antiporter (2.A.13.1.-) | Asuc_1999 Asuc_0142 |
| Aspartate/Fumarate | AspFumANTI | L-Aspartate [p] + Fumarate [c] <-> L-Aspartate [c] + Fumarate [p] | Antiporter (2.A.13.1.-) | Asuc_0142 |
| Fumarate | FumSipc | Fumarate [p] + Sodium [p] -> Fumarate [c] + Sodium [c] | Symporter (2.A.47.1.-) | Asuc_1568 |
| Fumarate | FumSpc | Fumarate [p] + H^+^ [p] -> Fumarate [c] + H^+^ [c] | Symporter (2.A.56.1.-) | Asuc_0146  Asuc_0147  Asuc_0148  Asuc_0156  Asuc_0157  Asuc_0158  Asuc_0270  Asuc_0271  Asuc_0272  Asuc_0366  Asuc_0367  Asuc_0368  Asuc_1163  Asuc_1164  Asuc_1165  Asuc_1577  Asuc_1578  Asuc_1579  Asuc_1921  Asuc_1922  Asuc_1923 |
| D-Galactarate | GalctSpc | D-Galactarate [p] + H^+^ [p] <-> D-Galactarate [c] + H^+^ [c] | Symporter (2.A.1.14.-) | Asuc_1584 |
| D-Glucarate | GlcrSpc | D-Glucarate [p] + H^+^ [p] <-> D-Glucarate [c] + H^+^ [c] | Symporter (2.A.1.14.-) | Asuc_1584 |
| Formate | Forfpc | Formate [p] <-> Formate [c] | Permease (1.A.16.2.-) | Asuc_0208 |
| Ions and metals | | | | |
| Chloride | ClANTI | 2.0 * Cl^-^ [p] + H^+^ [c] <-> 2.0 * Cl^-^ [c] + H^+^ [p] | Antiporter (2.A.49.5.-) | Asuc_0454 |
| Fe^2+^ | Fe2ABC | ATP [c] + Fe^2+^ (fe2)[p] + H_2_O [c] -> ADP [c] + Fe^2+^ [c] + Pi [c] | ABC transporter (3.A.1.20.-, 3.A.1.15.-) | Asuc_1682  Asuc_1683  Asuc_1684  Asuc_1715  Asuc_1716  Asuc_1717  Asuc_1820  Asuc_1821  Asuc_1822  Asuc_1823 |
| Fe^2+^ | Fe2fpc | Fe^2+^ [p] -> Fe^2+^ [c] | Permease (9.A.8.1.-) | Asuc_1014 |
| Fe^3+^ | Fe3ABC | ATP [c] + Fe^3+^ [p] + H_2_O [c] -> ADP [c] + Fe^3+^ [c] + Pi [c] | ABC transporter (3.A.1.10.-, 3.A.1.14.-) | Asuc_1017  Asuc_1018  Asuc_1019  Asuc_1020  Asuc_1682  Asuc_1683  Asuc_1684  Asuc_1715  Asuc_1716  Asuc_1717  Asuc_1820  Asuc_1821  Asuc_1822  Asuc_1823 |
| Thiosulfate | TsulABC | ATP [c] + H_2_O [c] + Thiosulfuric acid [p] -> ADP [c] + Pi [c] + Thiosulfuric acid [c] | ABC transporter (3.A.1.6.-) | Asuc_1692  Asuc_1693  Asuc_1694  Asuc_1695 |
| Sulfate | SO4ABC | ATP [c] + H_2_O [c] + Sulfate [p] -> ADP [c] + Pi [c] + Sulfate [c] | ABC transporter (3.A.1.6.-) | Asuc_1692  Asuc_1693  Asuc_1694  Asuc_1695 |
| Sulfate | SO4Spc | H^+^ [p] + Sulfate [p] -> H^+^ [c] + Sulfate [c] | Symporter (2.A.53.3.-) | Asuc_2058  Asuc_1199 |
| Potassium | Kfcp | K^+^ [c] -> K^+^ [p] | Permease (1.A.23.1.-) | Asuc_0965 |
| Potassium | KSpc | H^+^ [p] + K^+^ [p] -> H^+^ [c] + K^+^ [c] | Symporter (2.A.38.1.-) | Asuc_0246  Asuc_0512 |
| Potassium | Kfpc | K^+^ [p] -> K^+^ [c] | Symporter (9.A.4.1.-) | Asuc_1978 |
| Potassium/Zinc | KZnANTI | H^+^ [p] + K^+^ [p] + Zn^2+^ [c] -> H^+^ [c] + K^+^ [c] + Zn^2+^ [p] | Antiporter (2.A.4.1.3) | Asuc_0551  Asuc_1517 |
| Zinc | ZnABC | ATP [c] + H_2_O [c] + Zn^2+^ [p] -> ADP [c] + Pi [c] + Zn^2+^ [c] | ABC transporter (3.A.1.15.-) | Asuc_1826  Asuc_1825  Asuc_1782 |
| Zinc | Znfpc | Zn^2+^ [p] -> Zn^2+^ [c] | Permease (2.A.5.5.-) | Asuc_0416 |
| Sodium | NaHANTI | 2.0 * H^+^ [p] + Sodium [c] <-> 2.0 * H^+^ [c] + Sodium [p] | Antiporter (2.A.35.2.-) | Asuc_1886  Asuc_1995 |
| Cobalt | Co2fpc | Co^2+^ [p] -> Co^2+^ [c] | Permease (1.A.35.1.-) | Asuc_1979 |
| Cobalt | Co2HANTI | Co^2+^ [c] + H^+^ [p] -> Co^2+^ [p] + H^+^ [c] | Antiporter (2.A.4.1.-) | Asuc_0551  Asuc_1517 |
| Cobalt | Co2fcp | Co^2+^ [c] -> Co^2+^ [p] | Permease (9.A.40.1.-) | Asuc_1919 |
| Molybdate | MobdABC | ATP [c] + H_2_O [c] + Molybdate [p] -> ADP [c] + Molybdate [c] + Pi [c] | ABC transporter (3.A.1.8.-, 3.A.1.6.8) | Asuc_0181  Asuc_0180  Asuc_1559  Asuc_1560 |
| Magnesium | Mg2fpc | Mg^2+^ [p] <-> Mg^2+^ [c] | Permease (1.A.35.1.-) | Asuc_1979 |
| Phosphate | PiSipc | Sodium [p] + Pi [p] -> Sodium [c] + Pi [c] | Permease (2.A.20.2.-) | Asuc_1077 |
| Phosphate | PiSpc | H^+^ [p] +Pi [p] -> H^+^ [c] + Pi [c] | Permease (2.A.20.2.-) | Asuc_1077 |
| Nitrite/Nitrate | No3No2ANTI | Nitrite [c] + Nitrate [p] -> Nitrite [p] + Nitrate [c] | Antiporter | Unknown |
| **Vitamins and others** | | | | |
| Ascorbate | AscPTS | Ascorbate [p] + Phosphoenolpyruvate [c] -> L-Ascorbate 6-phosphate [c] + Pyruvate [c] | PTS (4.A.7.1.1) | Asuc_0238  Asuc_0239 |
| Nicotinamide-beta-riboside | RNAMtpp | Nicotinamide-beta-riboside [p] -> Nicotinamide-beta-riboside [c] | Permease (4.B.1.1.2) | Asuc_1993 |
| Thiamin | ThmABC | ATP [c] + H_2_O [c] + Thiamin [p] -> ADP [c] + Pi [c] + Thiamin [c] | ABC transporter  (3.A.1.19.-) | Asuc_1129  Asuc_1130  Asuc_1131 |
| Thiamin monophosphate | ThmmpABC | ATP [c] + H_2_O[c] + Thiamin monophosphate [p] -> ADP [c] +Pi [c] + Thiamin monophosphate [c] | ABC transporter  (3.A.1.19.-) | Asuc_1129  Asuc_1130  Asuc_1131 |
| Thiamin diphosphate | ThmppABC | ATP [c] + H_2_O [c] + Thiamin diphosphate [p] -> ADP [c] + Pi [c] + Thiamin diphosphate [c] | ABC transporter  (3.A.1.19.-) | Asuc_1129  Asuc_1130  Asuc_1131 |
| Putrescine | PtrcABC | ATP [c] + H_2_O [c] + Putrescine [p] -> ADP [c] +Pi [c] + Putrescine [c] | ABC transporter  (3.A.1.11.-) | Asuc_1743  Asuc_1744  Asuc_1745  Asuc_1746  Asuc_1525  Asuc_1526  Asuc_1527  Asuc_1528 |
| Spermidine | SpmdABC | ATP [c] + H_2_O [c] + Spermidine [p] -> ADP [c] + Pi [c] + Spermidine [c] | ABC transporter  (3.A.1.11.-) | Asuc_1743  Asuc_1744  Asuc_1745  Asuc_1746  Asuc_1525  Asuc_1526  Asuc_1527  Asuc_1528 |
| Heme | PhemeABC | ATP [c] + H_2_O [c] + Heme [p] -> ADP [c] + Heme [c] +Pi [c] | ABC transporter  (3.A.1.5.2, 3.A.1.14.11, 3.A.1.14.21, 3.A.1.14.10, 3.A.1.14.16, 3.A.1.14.19, 3.A.1.14.18) | Asuc_0903  Asuc_0904  Asuc_0905 |
| Heme | PhemeABCp | ATP [c] + H_2_O [c] + Heme [c] -> ADP [c] + Heme [p] +Pi [c] | ABC transporter  (3.A.1.5.2, 3.A.1.14.11, 3.A.1.14.21, 3.A.1.14.10, 3.A.1.14.16, 3.A.1.14.19, 3.A.1.14.18) | Asuc_0217  Asuc_1489 |
| Pantothenate | PntoSpc | Sodium [p] + Pantothenate [p] -> Sodium [c] + Pantothenate [c] | Symporter  (2.A.21.1.-) | Asuc_0645 |
| Cytosine | CsnSpc | Cytosine [p] + H^+^ [p] -> Cytosine [c] + H^+^ [c] | Symporter  (2.A.39.1.-) | Asuc_1189 |
| Adenine | AdeSpc | Adenine [p] + H^+^ [p] -> Adenine [c] + H^+^ [c] | Symporter  (2.A.40.7.-) | Asuc_1180 |
| Uracil | UraSpc | H^+^ [p] + Uracil [p] -> H^+^ [c] + Uracil [c] | Symporter  (2.A.40.1.-) | Asuc_1772 |
| Xanthine | XanSpc | H^+^ [p] + Xanthine [p] -> H^+^ [c] + Xanthine [c] | Symporter  (2.A.40.4.-) | Asuc_1902 |
| Hypoxanthine | Hxanfpc | Hypoxanthine [p] <-> Hypoxanthine [c] | Permease  (2.A.40.7.-) | Asuc_0940 |
| Guanine | Guafpc | Guanine [p] <-> Guanine [c] | Permease  (2.A.40.7.-) | Asuc_0940 |
| Cytidine | CytdSpc | Cytidine [p] + H^+^ [p] <-> Cytidine [c] + H^+^ [c] | Symporter  (2.A.1.10.-) | Asuc_0358 |
| Inosine | InsSpc | H^+^ [p] + Inosine [p] <-> H^+^ [c] + Inosine [c] | Symporter  (2.A.1.10.-) | Asuc_0358 |
| Thymidine | ThymdSpc | H^+^ [p] + Thymidine [p] <-> H^+^ [c] + Thymidine [c] | Symporter  (2.A.1.10.-) | Asuc_0358 |
| Adenosine | AdnSpc | Adenosine [p] + H^+^ [p] <-> Adenosine [c] + H^+^ [c] | Symporter  (2.A.1.10.-) | Asuc_0358 |
| Xanthosine | XtsnSpc | H^+^ [p] + Xanthosine [p] <-> H^+^ [c] + Xanthosine [c] | Symporter  (2.A.1.10.-) | Asuc_0358 |
| **Amino acids** | | | | |
| L-Tryptophan | TrpSpc | H+ [p] + L-Tryptophan [p] -> H+ (h)[c] + L-Tryptophan (trp-L)[c] | Symporter  (2.A.75.1.-) | Asuc_0628 |
| L-Tryptophan | TrpABC | ATP [c] + H_2_O [c] + L-Tryptophan [p] -> ADP [c] + Pi [c] + L-Tryptophan [c] | ABC transporter  (3.A.1.3.-) | Asuc_1948  Asuc_1949  Asuc_1950  Asuc_1951 |
| L-Glutamine | GlnABC | ATP [c] + L-Glutamine [p] + H_2_O [c] -> ADP [c] + L-Glutamine [c] + Pi [c] | ABC transporter  (3.A.1.3.-) | Asuc_1948  Asuc_1949  Asuc_1950  Asuc_1951 |
| L-Threonine | ThrScp | H^+^ [p] + L-Threonine [c] -> H^+^ [c] + L-Threonine [p] | Antiporter  (2.A.76.1.-) | Asuc_1484 |
| L-Threonine | ThrABC | ATP [c] + H_2_O [c] + L-Threonine [p] -> ADP [c] + Pi [c] + L-Threonine [c] | ABC transporter  (3.A.1.3.-) | Asuc_1948  Asuc_1949  Asuc_1950  Asuc_1951 |
| L-Threonine | ThrSpc | Sodium [p] + L-Threonine [p] -> Sodium [c] + L-Threonine [c] | Symport  (2.A.23.4.-) | Asuc_0947 |
| L-Serine | SerSpc | Sodium [p] + L-Serine [p] -> Sodium [c] + L-Serine [c] | Symport  (2.A.23.4.-) | Asuc_0947 |
| L-Serine | SerABC | ATP [c] + H_2_O [c] + L-Serine [p] -> ADP [c] + Pi [c] + L-Serine [c] | ABC transporter  (3.A.1.4.3, 3.A.1.3.18, 3.A.1.4.6, 3.A.1.4.2, 3.A.1.3.8) | Asuc_1948  Asuc_1949  Asuc_1950  Asuc_1951 |
| Glycine | GlyNaSpc | Glycine [p] + Sodium [p] -> Glycine [c] + Sodium [c] | Symporter  (2.A.22.1.-) | Asuc_0284 |
| Glycine | GlyClSpc | Cl^-^ [p] + Glycine [p] -> Cl^-^ [c] + Glycine [c] | Symporter  (2.A.22.1.-) | Asuc_0284 |
| L-Isoleucine | IleABC | ATP [c] + H_2_O [c] + L-Isoleucine [p] -> ADP [c] + L-Isoleucine [c] +Pi [c] | ABC transporter  (3.A.1.4.3, 3.A.1.3.18, 3.A.1.4.1, 3.A.1.3.8) | Asuc_1948  Asuc_1949  Asuc_1950  Asuc_1951 |
| L-Isoleucine | IleANTI | H^+^ [p] + L-Isoleucine [c] -> H^+^ (h)[c] + L-Isoleucine [p] | Antiporter  (2.A.78.1.-) | Asuc_1679  Asuc_1678 |
| L-Arginine | ArgScp | L-Arginine [p] + H^+^ [p] -> L-Arginine [c] + H^+^ [c] | Symporter  (2.A.75.1.-) | Asuc_0217 |
| L-Arginine | ArgABC | L-Arginine [p] + ATP [c] + H_2_O [c] -> ADP [c] + L-Arginine [c] + Pi [c] | ABC transporter  (3.A.1.3.-) | Asuc_0298  Asuc_0299  Asuc_0300  Asuc_1807  Asuc_0617  Asuc_0618  Asuc_0619  Asuc_0620 |
| L-Lysine | LysScp | H^+^ [p] + L-Lysine [p] -> H^+^ [c] + L-Lysine [c] | Symporter  (2.A.75.1.-) | Asuc_0217 |
| L-Cysteine | CysABC | ATP [c] + L-Cysteine [p] + H_2_O [c] -> ADP [c] + L-Cysteine [c] + Pi [c] | ABC transporter  (3.A.1.3.-) | Asuc_0298  Asuc_0299  Asuc_0300  Asuc_1807 |
| L-Methionine | MetABC | ATP [c] + H_2_O [c] + L-Methionine [p] -> ADP [c] + L-Methionine [c] + Pi [c] | ABC transporter  (3.A.1.4.-) | Asuc_0034  Asuc_0033  Asuc_0032 |
| L-Methionine | Metfpc | L-Methionine[p] <-> L-Methionine [c] | Permease (2.A.95.1.-) | Asuc_0594 |
| L-Glutamate | GluLABC | ATP [c] + L-Glutamate [p] + H_2_O [c] -> ADP [c] + L-Glutamate [c] + Pi [c] | ABC transporter  (3.A.1.4.-, 3.A.1.3.-) | Asuc_1948  Asuc_1949  Asuc_1950  Asuc_1951 |
| L-Glutamate | GluLSpc | L-Glutamate [p] + Sodium [p] -> L-Glutamate [c] + Sodium [c] | Symporter  (2.A.27.1.-) | Asuc_0452 |
| L-Glutamate | GluLSHpc | L-Glutamate [p] + H^+^ [p] -> L-Glutamate [c] + H^+^ [c] | Symporter (2.A.75.1-) | Asuc_0217 |
| D-Glutamate | GluDSpc | D-Glutamate [p] + Sodium [p] -> D-Glutamate [c] + Sodium [c] | Symporter  (2.A.27.1.-) | Asuc_0452 |
| L-Tyrosine | TyrABC | ATP [c] + H_2_O [c] + L-Tyrosine [p] -> ADP [c] +Pi [c] + L-Tyrosine [c] | ABC transporter  (3.A.1.4.-, 3.A.1.3.-) | Asuc_1948  Asuc_1949  Asuc_1950  Asuc_1951 |
| L-Alanine | AlaABC | L-Alanine [p] + ATP [c] + H_2_O [c] -> ADP [c] + L-Alanine [c] +Pi [c] | ABC transporter  (3.A.1.3.-) | Asuc_1948  Asuc_1949  Asuc_1950  Asuc_1951 |
| L-Phenylalanine | PheABC | ATP [c] + H_2_O [c] + L-Phenylalanine [p] -> ADP [c] + L-Phenylalanine [c] + Pi [c] | ABC transporter  (3.A.1.3.-) | Asuc_1948  Asuc_1949  Asuc_1950  Asuc_1951 |
| L-Asparagine | AsnABC | L-Asparagine [p] + ATP [c] + H_2_O [c] -> ADP [c] + L-Asparagine [c] + Pi [c] | ABC transporter  (3.A.1.3.-) | Asuc_1948  Asuc_1949  Asuc_1950  Asuc_1951 |
| L-Histidine | HisABC | ATP [c] + H_2_O [c] + L-Histidine [p] -> ADP [c] + L-Histidine [c] + Pi [c] | ABC transporter  (3.A.1.3.-) | Asuc_1948  Asuc_1949  Asuc_1950  Asuc_1951 |
| L-Leucine | LeuABC | ATP [c] + H_2_O [c] + L-Leucine [p] -> ADP [c] + L-Leucine [c] + Pi [c] | ABC transporter  (3.A.1.3.-) | Asuc_1948  Asuc_1949  Asuc_1950  Asuc_1951 |
| L-Proline | ProABC | ATP [c] + H_2_O [c] + L-Proline [p] -> ADP [c] + Pi [c] + L-Proline [c] | ABC transporter  (3.A.1.3.-) | Asuc_1948  Asuc_1949  Asuc_1950  Asuc_1951 |
| L-Valine | ValABC | ATP [c] + H_2_O [c] + L-Valine [p] -> ADP [c] + Pi [c] + L-Valine [c] | ABC transporter  (3.A.1.3.-) | Asuc_1948  Asuc_1949  Asuc_1950  Asuc_1951 |
