# Supplementary material for: Reconstruction of a genome-scale metabolic model for Actinobacillus succinogenes 130Z
Source: BMC Syst Biol. 2018 May 30;12:61. doi: 10.1186/s12918-018-0585-7 (PMC5975692; doi:10.1186/s12918-018-0585-7)
Supplement: Supplementary file 5 — Growth predictions on glycerol with and without DMSO. (DOCX 21 kb) [file 12918_2018_585_MOESM5_ESM.docx]

Additional file 5: Growth predictions on glycerol with and without DMSO

Figure SI 1. Biomass yields on glycerol when increasing the uptake rate of dimethyl sulfoxide (DMSO).
